# Shadow Effect for Small Insect Detection by W-Band Pulsed Radar

**DOI:** 10.3390/s23229169

**Published:** 2023-11-14

**Authors:** Miguel Hernández Rosas, Guillermo Espinosa Flores-Verdad, Hayde Peregrina Barreto, Pablo Liedo, Leopoldo Altamirano Robles

**Affiliations:** 1 Electronics Department, National Institute of Astrophysics, Optics and Electronics, Sta. Ma. Tonantzintla, Puebla 72840, Mexico; mhdzr@inaoep.mx (M.H.R.); gespino@inaoep.mx (G.E.F.-V.); 2 Computational Sciences Department, National Institute of Astrophysics, Optics and Electronics, Sta. Ma. Tonantzintla, Puebla 72840, Mexico; hperegrina@inaoep.mx; 3 Arthropod Ecology and Pest Management Deparment, El Colegio de la Frontera Sur, Tapachula 30700, Mexico; pliedo@ecosur.mx

**Keywords:** radar, entomology, shadow effect, W-band radar, fruit fly, insect detection, radar cross-section, RCS, radar entomology, Mediterranean fruit fly, pulsed radar

## Abstract

In radar entomology, one primary challenge is detecting small species (smaller than 5 cm) since these tiny insects reflect radiation that can be poorly observable and, therefore, difficult to interpret. After a literature search on radar entomology, this research found few works where it has been possible to sense insects with dimensions smaller than 5 cm using radars. This paper describes different methodologies to detect Mediterranean fruit flies with 5–6 mm sizes using a pulsed W-band radar and presents the experimental results that validate the procedures. The article’s main contribution is the successful detection of Mediterranean fruit flies employing the shadow effect on the backscattered radar signal, achieving an 11% difference in received power when flies are present. So far, according to the information available and the literature search, this work is the first to detect small insects less than 1 cm long using a pulsed radar in W-Band. The results show that the proposed shadow effect is a viable alternative to the current sensors used in smart traps, as it allows not only detection but also counting the number of insects in the trap.

## 1. Introduction

The radar operation is relatively simple: a radio wave is transmitted by an antenna, and some waves are scattered by the object of interest (echo). They are captured back by the same antenna (or another antenna, depending on the radar type) to be processed [1]. The information about the target can be inferred from the difference between the signal that is transmitted–received. The information it provides depends on the design of the radar; however, this can often include distance, direction, speed, and even the target size [2]. The radar allows the detection of these variables using *remote sensing*; that is, applying techniques that acquire measurements from an object using an instrument far from the target [3]. For the radar, the information is present in the intensity, frequency, phase, and modulation of the radio signal; however, the main problem is their interpretation. It is not easy because it is necessary to determine how the properties of the object (for example, temperature) are translated by the characteristics of the receiver (for example, the wavelength of the object’s light) [4].

In a general classification, there are two types of radars, coherent and non-coherent [5]. Non-coherent radars are characterized by not preserving the phase information contained in the return signal; therefore, the detection is carried out fundamentally based on the amplitude. Non-coherent receivers are often found in simple radar systems since they do not require complicated hardware and software. Coherent radars have a known amplitude and phase for signal processing, such as pulse compression, Doppler processing, mono-pulse comparison, moving target indication, synthetic aperture radar imaging, and adaptive processing space-time [2]. These types of radars are more complex but also allow for obtaining more information about the target.

According to the operation frequency, radars are denoted with a letter set because radars were historically developed for military use. The IEEE (Institute of Electrical and Electronics Engineers) has adopted the band as a letter set. Table 1 shows the spectrum associated with each letter set. The UHF band is used for very long early warning systems (EWRs) for detecting and tracking satellites or ballistic missiles. The primary applications for L-band and S-band radars are ground-based and ship-based systems. The C-band is used for military surveillance, missile control, and ground surveillance. The X-band has applications in missile guidance and airborne imaging. The Ka-band is applied to avoid vehicle collisions, police traffic radar, and security monitor detectors. Both radars in V- and W-bands suffer from atmospheric attenuation, so their use is limited to the short range, with primary applications in the automotive industry for parking assistance or blind spot detection. Radar systems above this band are considered in *Tera Hertz* frequency and are an emerging technology [5].

Radar is the most popular technique among remote sensing solutions [3] because the information is present in the radar signal’s intensity, frequency, phase, and modulation [2]. Compared to other remote sensing techniques, such as infrared or ultrasound, radar is, most of the time, not sensitive to light, color, sound, acoustic noise, or temperature, as shown in Table 2.

Radar has been employed in insect studies since the 1960s; this application is known as *radar entomology*. Conventional entomological radars are non-coherent, and the signal amplitude and polarization information received from the insects are used to detect the density and class of aerial insects [6]. This type of radar operates at a wavelength of 3.2 cm in the X-band and uses a parabolic antenna with a beam width of one to two degrees. Coherent radars offer better measurement accuracy [3]. With this type of radar, both amplitude and phase information can be used to extract the micro-Doppler frequency induced by the vibratory components of the target. Coherent radars operating the W band achieve the best performance in measurement precision and the smallest measurable size of the insect [4].

Particular challenges that emerge when the radar is applied in entomology are primarily associated with issues originating from the backscattered signal. These drawbacks include, among others, intensity decay, absorption (or complete blockage), scattering (additional reflections), refraction (change of direction caused by the shift in medium), and drift (caused by lateral movements of the medium). The measurement context is another variable that produces problems with radars, for example, when insects settle or crawl on vegetation surfaces. However, this problem can be solved with visual methods, such as image analysis. Despite these limitations, radar continues to be one of the most effective tools for observing insect flight because it works in severe weather conditions and provides round-the-clock observations [4].

In the case of radar entomology, it can focus on the individual or group level, on aspects such as migration, behavior, target characterization, and surveillance [4]. For these applications, it is customary to use radars of type scanning, vertical looking (VLR), harmonics, or frequency-modulated continuous wave (FMCW) [7].

The signals provided by the radars are used to estimate the parameters of an isolated insect [6]. For example, to calculate the mass of an insect, in [8] the authors proposed in 1989 to use the radar cross-section of the insect (RCS) since it was observed that there was a logarithmic relationship between the mass of the insect and its average RCS [8]. The RCS quantifies the target’s backscatter and characterizes how much of the transmitted signal was intercepted by the target; it depends not only on the material’s properties but also on the frequency, polarization, orientation, geometry, and angle of incidence of the transmitted wave [9].

Another parameter that can be determined using the radar is the target’s orientation, which, according to Long [6], can be calculated using the polarization pattern; the power of the received signal reaches its maximum value when the polarization direction is parallel to the axis of the insect’s body. This principle has been shown to perform well for *vertical-looking* type radars (VLRs). The wing beating is another parameter that can be calculated using radars, such as radars in the W band. The wing beating causes a fluctuation in the amplitude of the radar signal, producing a periodic modulation and, therefore, it can be used to detect and characterize the wing beating [10].

Insect tracking is another radar application; it is carried out using the *harmonic radar* technique, which consists of attaching a small transponder to the insect and exciting it with the radar to re-emit a new signal at a different frequency [11]. This application has been widely used to study the movement of insects in plantations, such as the study of the displacement of the melon flies, *Zeugodacus cucurbitae*, which are major pests that pose invasion risks [12], or *Vespa velutina*, which preys on pollinating insects [13]. For honeybee surveillance, Nawaf et al. [14] used a 5.8 GHz continuous-wave radar to monitor free-flying activity. Using machine learning techniques, the authors were able to automate the classification of different activities, such as leaving, entering, and hovering.

For insect migration and early detection, the *vertical-looking* radar (VLR) is used. For example, Chapman et al. [15] used a VLR to monitor migrant insect populations. Rotating the polarization of the radar and nutation, Chapman et al. detected different insects in the air at different altitudes. A migration flux database was created for different species. In a similar work, Wang et al. [16] also used a VLR to detect different insects in the air but focused on ascent and descent behaviors. Without specifying the species, the author was able to detect insects weighing less than 10 mg.

A big problem with radars is related to measurements; small insects reflect radiation that can be hardly observable and, therefore, tricky to interpret. This condition is especially true when the dimensions of the insect are around or less than 5 cm [17]. The measurements are influenced not just by size but also by the target’s aspect and its orientation relative to the antenna, which directly impacts a small RCS magnitude [4]. Another factor affecting the RCS is humidity, which is a main reflective component. In 1966, Hajovsky et al. [18] reported that the loss of body moisture affects the dispersal properties of insects, noting a significant decrease in humidity after their death.

Several studies have focused on measuring insects with a size dimension smaller than 5 cm. Rui et al. [19] used X-band and Ku-/K-band radars to detect different types of moths. Using the RCS, the authors were able to classify the mass and body length. For mass estimation, the authors achieved double the precision compared with traditional methods. For body lengths, the authors measured body length from 6 to 28 mm using a range of frequencies, from 4 to 38 GHz. A similar study was conducted by Wang [20], where the use of support vector regression (SVR) was proposed to estimate the mass of different moth species. In the experiment, the authors used an X-band radar and a microwave anechoic chamber, where the moth was attached to a suspended polyethylene line. The sizes of the different species were not reported.

Continuing with body width and length estimations, Li et al. [21] used an X-band polarimetric radar to estimate the body widths and lengths of different insects. The authors took 159 insects of different sizes and split them into three groups. For small insects, sizes ranged from 10 to 20 mm; medium insects had sizes between 20 and 30 mm, while large insects ranged from 30 to 47 mm. They proposed empirical equations based on the different RCS parameters to estimate the body widths and lengths of the insects.

Riley [22] used an X-band radar and a transmission line to prevent beam scattered; this allowed measuring tiny insects (less than 1 cm). In a study conducted by Wang et al. [10], the authors measured different targets of the *Mythimna separata* species, ranging from 10–42 mm, using FMCW radars in the W and S bands. For monitoring free-flying insects, Diyap et al. [23] used a continuous wave radar in the W-band to detect two species: mosquitoes (*Culex pipiens*) and bees (*Apis mellifera*). In both cases, due to the dimensions of the insects, the authors used the *micro-Doppler* effect generated by wing beats to detect and classify the species. Simulation and experimental results validated the proposed method.

Table 3 shows a summary of the different studies reported in the literature. As observed, these studies primarily focus on estimating insect size, mass, body width, length, surveillance, and tracking. Also, the equipment setup for insect measurements tends to be bulky and not easily portable. In this sense, studies have reported the use of anechoic chambers and heavy equipment. The insect’s size is not reported in all studies but the smallest insect reported was 10 mm. Regarding the species, the most common insects are moths and bees. The most common radars used are the X-band, Ku-band, and W-band. For the radar type, FMCW, VLR, and CW are mainly used.

According to the literature, the present investigation is the first to detect a Mediterranean fruit fly with a W-band pulsed radar. This species is considered one of the most destructive agricultural pests in the world, capable of causing serious losses if not controlled [24]. The utilization of the W-band radar remains uncommon in entomology; however, the findings presented in this paper create new prospects for applying this technology to detect small insects. The proposal shows the potential of using the radar for counting, which is new in radar entomology literature. Regarding *smart traps*, we found no reports on the use of radars [25,26,27,28]. Thus, the present research will be the first proposal and validation for its implementation.

## 2. Materials and Methods

In the initial experiment, a pulsed W-band radar from Acconer© was used to measure Mediterranean fruit flies, Ceratitis capitata (Wied.), which are insects that are approximately 5–6 mm in size. The sterile flies were provided as pupae by the Mediterranean Fruit Fly Rearing and Sterilization facility (SENASICA), located in Metapa, Chiapas, Mexico. After emergence, the flies were fed sugar and water separately.

**Figure 1:**
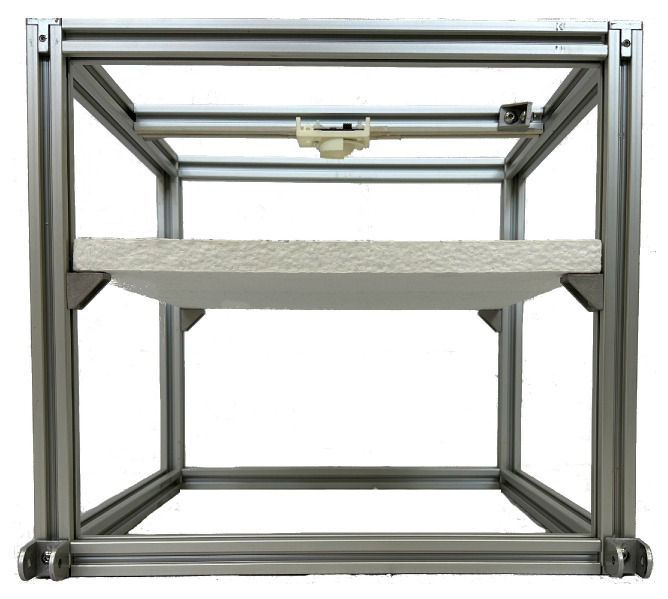
Experimental arrangement to fix the radar for the detection of insects.

The experiment’s arrangement is shown in Figure 1. The radar was placed in the center of a structured aluminum cube with dimensions of 30 × 30 × 30 cm, pointing toward its base. The cube’s walls were constructed with transparent acrylic to prevent flies from escaping, and the cube had a door to access the inside. A 2 cm thick polystyrene base was used to place the target, providing mechanical stability and preventing the radar from being affected by the material’s reflectivity. The polystyrene base was used because it is a material that does not affect the signal received by the radar since it has low reflectivity [10].

The W-band pulsed radar used was the Acconeer©A111, with an operating frequency of 60.5 GHz and a spatial resolution of 5 mm. Covering a distance of 30 cm, the radar will transmit 600 pulses to sweep the distance range and generate the corresponding data points. The radar provides two primary *services*: the *envelope service* and the *IQ service*. The envelope service provides the envelope of the received signal, which is the amplitude of the received signal as a function of distance. The IQ service provides the in-phase and quadrature components of the received signal. They can be used to compute the amplitude and phase. Only the envelope service was used in this research.

The following parameters were used to configure the radar. The first parameter was antenna gain, which can be adjusted between 0.0 and 1.0. The recommended value for this parameter is 0.5; however, for the proposed experiments, measurements began at 0.1, and the value was incrementally increased to prevent saturation of the ADC converter. Saturation occurs when the reflectiveness of the material is too high with the gain set, so the API will automatically detect this condition; according to the documentation provided, the ADC’s gain should be reduced to have the right measurement. The second parameter was the hardware acceleration average sample (HWAAS), i.e., the number of averaged samples used to obtain a single measurement. This parameter is used to reduce the signal noise. For this experiment, an initial value of 15 was used, which is the initial value recommended by the radar manufacturer. The third parameter was the power save mode, which reduces the radar’s power consumption. For this experiment, it was disabled to obtain the best possible performance. The fourth parameter was the update rate, which is the number of frames obtained per second. A frame is just the data for a complete azimuthal sweep, up to a maximum range. For this experiment, the default value of 30 Hz was used. The fifth parameter was the profile, which controls the length of sent pulses and how they are sampled upon return. The max resolution profile was used for the experiments, which is recommended for close ranges (less than 20 cm). Finally, the sixth parameter was the range set between 10 and 26 cm. This parameter controls the range of distances at which the measurements are taken.

One thousand sweeps were performed to obtain a single final measurement; they were then averaged to minimize the impact of noise. This procedure for noise diminishing is justified because the object is at a fixed distance, and the noise where the signal is immersed has zero mean. Thus, this will help to avoid errors with the interpretation of object distance. All the experiments were conducted with this condition.

Based on the works reported by Wang [10] and Riley [22], the first experiment did not use lenses or any other radar accessories. However, it was found that the antenna gain was low and dispersed; thus, according to Riley’s [22] proposal, this led to adding a lens to improve the antenna gain and better concentrate the signal beam. The lenses used were from Acconeer: the Fresnel (FZP) and a hyperbolic lens (HBL), both made of solid polyamide PA12. Each lens is dependent on its position on the *printed circuit board* holder (PCB). Table 4 summarizes the gain and angle (in the horizontal and vertical planes) for the signal beam, based on the lens used.

**Figure 2:**
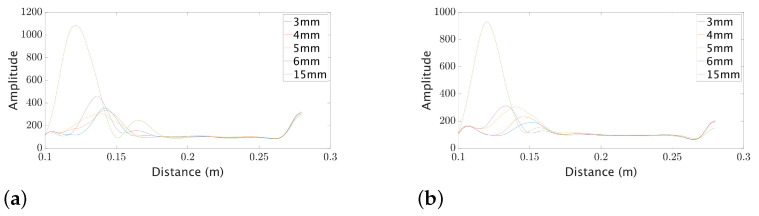
Results of the measured intensities for spheres of different sizes using the FZP (graph **a**) and HBL (graph **b**) lenses.

The target was modeled using metal spheres of different sizes to characterize the radar and determine the presence of a target. According to Drake [4], metal spheres or water drops are the ideal models that can be used for insect representation due to their high reflectivity. For this purpose, metal spheres were placed in polystyrene support and at a distance of 13 cm from the radar (since the minimum distance suggested by the manufacturer is 10 cm). The used sphere sizes were 3, 4, 5, 6, and 15 mm in diameter. Figure 2 summarizes the obtained results for each sphere, reaching a minimum detection with a sphere of 3 mm.

*Radar detection* occurs when the received signal exceeds a particular threshold value. Three parameters are associated with the detection: probability of detection ($P_{d}$), probability of missing ($P_{m}$), and probability of false alarm ($P_{f}$). $P_{d}$ denotes the probability that the radar detects a target when it is present. $P_{m}$ denotes the probability that the radar does not detect a target when it is present. $P_{f}$ denotes the probability that the radar detects a target when it is not present. $P_{d}$ and $P_{m}$ are related to the radar’s sensitivity, and $P_{f}$ is related to the radar’s noise level. To increase $P_{d}$, the *signal-to-noise ratio* (SNR) must be increased. The SNR is the ratio of the signal power to the noise power. In the experiment, the noise level was measured without the presence of a target. Calculating the variance for noise level, it was observed that it was below 200 using the polystyrene base. With this condition, the threshold value was chosen to produce the results in Figure 2. This assumption is true due to the fact that the noise level is zero mean, and the variance is the square of the standard deviation.

**Figure 3:**
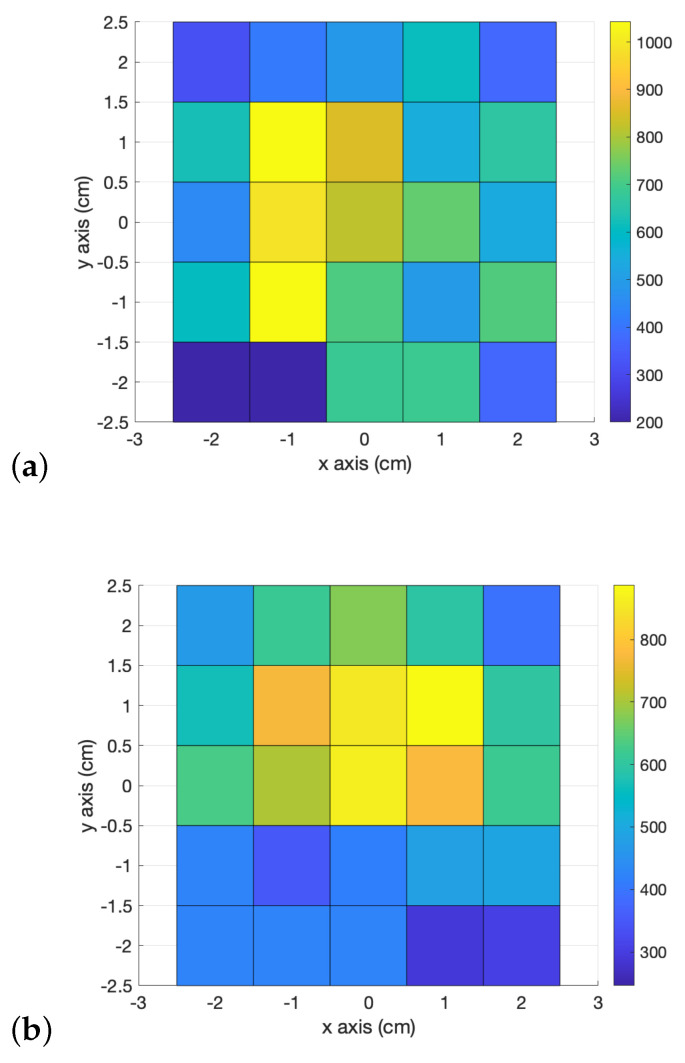
Radar intensity for FZP (**a**) and HBL (**b**) lenses. X and Y axes are in cm.

Another experiment used an identical metal sphere of 5 mm to continue with the radar characterization. For this purpose, a matrix of holes over a polystyrene base was used. Each hole had a diameter of 3 mm and a separation of 1 cm; all were drilled with a CNC machine to obtain good precision between holes and avoid misalignment between measurements. A 5 mm sphere was used as a target because this almost matched the medfly size and was placed in the orifices to determine the radar’s detection zone. The measurement ranges were in a 5 × 5 cm area, taking the center of the radar as a starting point (0, 0). One thousand frames were sampled and averaged for each hole to obtain a single measurement. Figure 3 shows the experimental results, wherein the X and Y axes represent the distances from the center of the radar, and the amplitude of the maximum received signal is in the Z axis.

**Figure 4:**
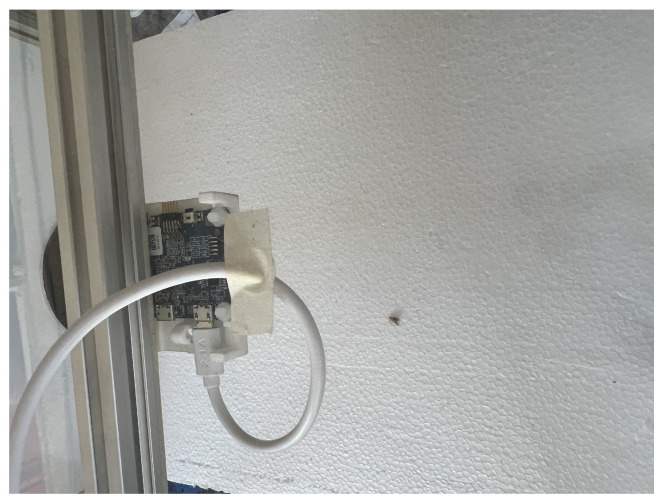
Radar experimental setup with the dead medfly.

**Figure 5:**
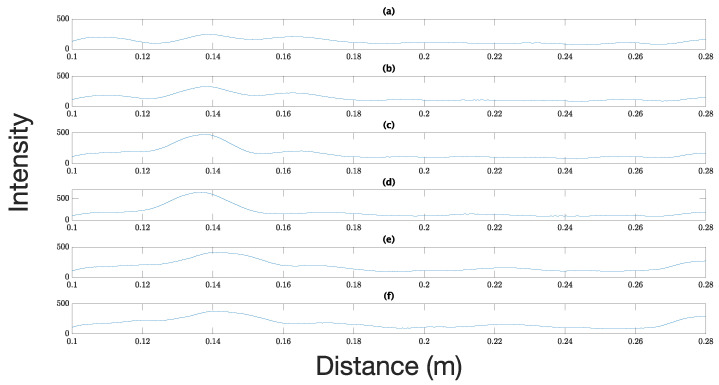
Radar detection for the Mediterranean fruit fly. Graphs (**a**,**b**) show the radar intensity before the detection threshold. Graphs (**c**,**d**) show the radar intensity after the detection threshold when the fly is in the detection zone. Note the increment in the intensity values by 0.14 m. Graphs (**e**,**f**) show the radar intensity after the detection when the fly moves away from the radar detection zone.

The results show that radar has a detection zone of 1.5 × 1.5 cm, close to the center. Here, it is important to note that when declaring the detection of a sphere, a threshold value was set to 200 after measuring the noise level of the radar (the same as in the first experiment), so the results over that value are considered as a sphere presence, and below that value, an absence. For absence values, those were normalized to 200 to visualize the results better. Once the detection of metal spheres and the minimum achievable resolution were validated, the same experiment was carried out with Mediterranean flies. Figure 4 shows the experimental setup for a medfly placed in the center of the base. In the beginning, dead flies were used to prevent movement; however, no detection was observed. This result was already reported by Drake [4]; it was corroborated since the dead flies did not present reflectivity due to the low moisture content of the dead bodies. Thus, live flies were used in the following experiments. These tests were carried out in the dark to avoid movement, but even under these conditions, the flies showed activity, which made detection difficult. Figure 5 shows that the medfly is detected by increasing the magnitude of the received signal that exceeds the detection threshold set at 200, which was determined by measuring the signal received in the open air.

**Figure 6:**
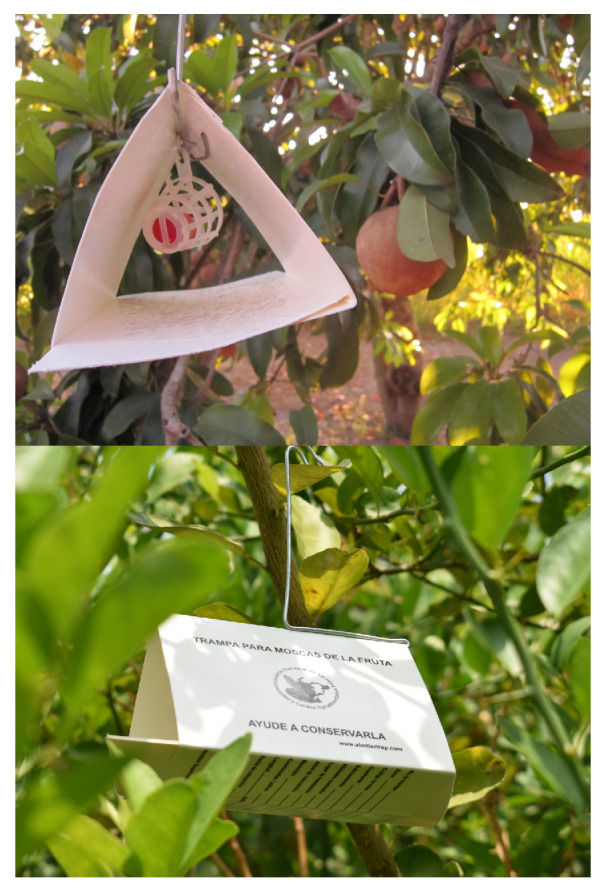
Delta trap used for medfly capture.

Given the difficulty of detecting live flies, another experiment was designed to validate the detection. According to Drake [4], carbon dioxide or cold is recommended for anesthetized live insects. In the experiment, the temperature of the flies was reduced to 4 °C for 3 min to loosen their movements. However, this was ineffective, as not all flies survived the low temperature. While detection was still possible, the received signal was weak. A new experiment was designed to solve the above problem and to validate the detection based on the field conditions in which the medfly was captured. For this, a literature search was carried out, and it was found that one of the primary medfly-capturing techniques involved using traps [29]. Although there are many traps, delta traps are primarily used in Mexico. This trap type has a triangular prism shape, with a glue base and a grid at the top where the attractant is placed. Figure 6 shows a delta-type trap. Therefore, it was decided to use the glue base as a reference point for a new detection experiment. For this, the same polystyrene base was used on which the glue base was placed. The same procedure was used for the metal spheres to measure the reflectivity of the glue base. Still, in this case, it was done with the two available lenses (FZP and HBL) and in different positions to vary the gain according to Table 4. It is crucial to characterize the glue base with the two lenses since the amount of glue is not uniform, and it affects the reflectivity obtained by the radar. In the experiment, the same glue base was used during all the measurements to avoid variations in the graphs presented. Once the bottom was characterized, flies were added to the glue base, and measurements were taken with the lenses. Two measurements were added with the variation of two parameters: HWAAS and gain. Measurements were made with the gain values of 0.1, 0.2, and 0.3. In the case of the HWAAS, values of 15, 30, and 40 were used. As a result, three measurements were made for each lens and positioned with the parameter variation mentioned above.

## 3. Results

**Figure 7:**
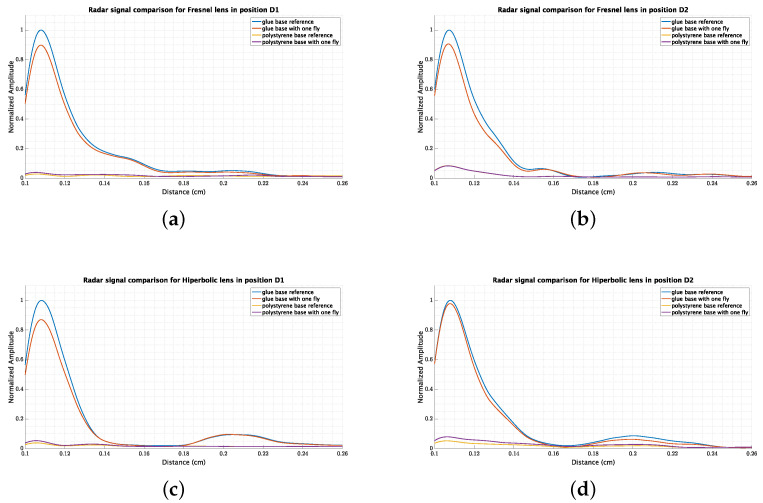
Results for radar measurements using polystyrene and glue bases.

Figure 7 shows the different measurements of the backscattered signal using different bases and with the presence of a fly. In these conditions, the fly causes an interference compared with the reference signal, and the *shadow effect* of the fly over the reference shows an improvement in the detection. For example, for the Fresnel lens (Figure 7a), the signal is 11% less than the reference, but much stronger than the signal with the polystyrene base.

**Figure 8:**
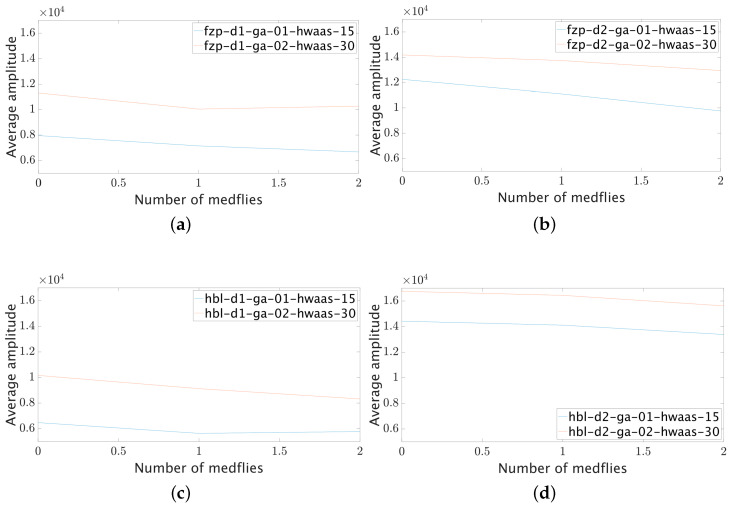
Results for medfly counting using different lens positions, gain values and HWAAS. Graphs (**a**,**b**) correspond to the FZP lens in position 1 and 2, respectively. Graphs (**c**,**d**) correspond to the HBL lens in position 1 and 2, respectively.

Figure 8a (top left corner) shows the graphs obtained for the FZP lens in position 1 with a gain of 0.1 and HWAAS of 15. It can be seen that the reflectivity of the glue base is the highest of all the measurements made, which corresponds to the expected result. Adding one and two flies decreased reflectivity. By doubling the gain of the amplifier and HWAAS to 0.2 and 30, respectively, the same behavior is observed. With a gain of 0.3 and HWAAS of 40, the radar’s ADC was saturated, so these results were not considered in this report.

Repeating the experiment for the FZP lens in position 2, the results are similar to those obtained in position 1; however, the reflectivity is higher due to the gain increase. Similarly, the data obtained for the gain of 0.3 and HWAAS of 40 were discarded due to saturation. The results are shown in Figure 8b (top right corner).

In the case of the HBL lens in position 1, the glue base reflectivity is lower than that obtained with the FZP lens, decreasing only up to the first fly, and slightly increasing for the second. When doubling the gain and HWAAS, the results are similar to those obtained with the FZP lens, as shown in Figure 8c (bottom left corner). The radar was saturated for a gain of 0.3 and HWAAS of 40, so these results were not considered. With the highest gain in position two and the HBL lens, the reflectivity increased above all measurements. Still, the results were maintained, where the reflectivity decreased for the second fly. These results held for a gain of 0.2 and HWAAS of 30, but the radar saturated for a gain of 0.3 and HWAAS of 40. The results are shown in Figure 8d (bottom right corner).

After the measurements were taken, a decreasing pattern was observed in all cases, so no more measurements were considered due to this pattern.

## 4. Discussion

Several experiments were conducted to detect medflies using a pulsed radar in the W band. These experiments included radar calibration in both reflectivity intensity and spatial distribution. The best configuration for medfly detection was the one that used a glue base characteristic of delta-type traps. We used the same glue base for all the experiments because changing it implies changing the background reflectivity and, therefore, the final measurements. It was possible to perceive the shadow effect, which helps to detect the medfly, despite the difference with the background reflectivity not being very high. Several measurements were conducted using different medflies sets, producing similar results. The simple average of the measurements is shown in Figure 7 and Figure 8. Based on the information provided in the figures, it can be inferred that medflies cause an 11% reduction in the backscattered radar signal compared to the signal without flies. This reduction, often called a shadow effect, demonstrates the effectiveness of pulsed radar in detecting medflies. Further experimentation is necessary to improve the rate of change in the backscattered signal, involving different glue bases and varying fly numbers for detection.

Additionally, a limitation of the radar system was observed in the form of a small detection zone, covering only a circular surface with a 1.5 cm radius. The corners of this detection area presented the most challenging regions for signal detection, characterized by weak signals and high noise levels. This behavior was consistent for both lenses and positions, resulting in variations in the achieved gain.

As discussed in Section 1, few works have been published on radar applications for small insect detection, so this research follows the results of Riley [22] and Wang [10], which propose experiments for insect detection using radars. To the best of our knowledge, this work is the first to use a pulsed radar in the W band to detect flies, especially medflies.

## 5. Conclusions

The radar proved to be an effective tool for detecting small objects modeled using spheres with a diameter of 3 mm. In this study, the radar was applied to detect insects, specifically medflies. We successfully detected medflies, which are 5–6 mm in length, using a pulsed radar in the W band with both FSL and HBL lenses. An approximately 11% *shadow effect* was observed in the backscattered radar signal, as evidenced by (Figure 7a). The detection of medflies was carried out using a glue base similar to a delta-type trap. The study demonstrated that the *radar cross-section* is well-suited for detecting small insects, such as those found on a glue base. Within the smart traps, this research opens up the possibility of using pulsed radar to detect and count insects. The proposed methodology is based on the conditions that insects are trapped in a glue base. So, this validates the radar for its application in smart traps and insect detection. Different works have been reported in the literature for the detection, classification, tracking, or parameter estimation of insects; however, this work is the first to use a commercial radar for detection. The radars reported in the literature are costly and not portable. This work demonstrates that a commercial radar can be used for the described task.

These results open up the possibility of using the cross-section of pulsed radars to identify different insect species, with each producing distinct shadow effects based on size and water content. It is important to note that further experimentation is required, including exploring different glue types, traps, and insect classes. Also, it is important to note that traps depend on the insects and conditions (material, lures, etc.) to capture different species. This work only focuses on the medfly, but initial results are promising for using radar to detect other insects following a similar approach.

## Figures and Tables

**Table 1:** Radar letter classification.

Letter Designation	Frequency Range in GHz (IEEE Standard)
HF	0.003–0.03
VHF	0.03–0.3
UHF	0.3–1.0
L-band	1.0–2.0
S-band	2.0–4.0
C-band	4.0–8.0
X-band	8.0–12.5
Ku-band	12.5–18.0
K-band	18.0–26.5
Ka-band	26.5–40.0
V & W or Millimeter Wave (MMW)	Normally > 34.0

**Table 2:** Comparison of remote sensing technologies.

	Sensibility			
Sensor	Temperature	Color	Light	Sound/Noise
Infrared	Yes	Yes	Yes	No
Ultrasonic	Yes	No	No	Yes
Radar	No	No	No	No

**Table 3:** Summary of the different studies reported in the literature.

	Radar			Insects	
Reference	Type	Band	Application	Specie	Dimensions
[10]	Coherent	W (93.6 GHz) and S (3.3 GHz)	Wing beats	Mythimna separata	10–42 mm
[11]	Harmonic	X (9.4 GHz)	Tracking	Vespa velutina	20 mm
[14]	CW	C (5.8 GHz)	Surveillance	Honeybees (no specie reported)	Not reported
[16]	VLR	Ku (16.2 GHz)	Detection and tracking	Different insects	Not reported
[19]	Multi-frequency	X (8.25–11.75 GHz), Ku (17.75–18 GHz) and K (18–23.75 GHz).	Morphological Parameter Estimation	Different moth species	6–28 mm
[20]	SFCW	X (9.4 GHz)	Mass estimation	Different moth species	Not reported
[21]	Polarimetric	X (9.4 GHz)	Width and length estimation	Insects from different species	10–47 mm
[22]		X		aphids and planthoppers	
[23]	CW	W (94.3 GHz)	Wing beat classification	mosquitoes (culex pipiens) and bees (apis mellifera)	Not reported
This work	Pulsed	W (60.5 GHz)	Detection and counting	Mediterranean fruit fly (Ceratitis capitata)	5–7 mm

**Table 4:** Gain reported by the manufacturer in the vertical (E-Plane) and horizontal (H-plane) planes, according to two lens positions on the holder (D1, D2). Source: manufacturer data sheet.

	Max. Gain (dBFS)		HPBW-E (degree)		HPBW-H (degree)	
	D1	D2	D1	D2	D1	D2
HBL	11.6	20	22	17	30	15
FZP	11.4	18.2	20	12	27	12

## Data Availability

The readings of the backscattered signals from the radar are available upon request to the correspondence author.

## Abbreviations

The following abbreviations are used in this manuscript:

ADC	analog to digital converter
CNC	computer numerical control
FMCW	frequency-modulated continuous wave
FZP	Fresnel lens
HBL	Hyperbolic lens
HF	high frequency
HPBW	half-power beamwidth
HWAAS	hardware-accelerated average sample
MMW	millimeter wave
PCB	printed circuit board
RCS	radar cross-section
UHF	ultra-high frequency
USDA	United States Department of Agriculture
VHF	very high frequency
VLR	vertical-looking radar
SNR	signal-to-noise ratio
